# Long-term cyclical *in vivo *loading increases cartilage proteoglycan content in a spatially specific manner: an infrared microspectroscopic imaging and polarized light microscopy study

**DOI:** 10.1186/ar2040

**Published:** 2006-09-06

**Authors:** Ehsan Saadat, Howard Lan, Sharmila Majumdar, David M Rempel, Karen B King

**Affiliations:** 1 Department of Bioengineering, University of California, Berkeley, 459 Evans Hall #1762 Berkeley, CA 94720-1762, USA; 2 Department of Radiology, University of California, San Francisco, 1700 4^th ^Street, Suite 203, Box 2520, San Francisco, CA 94107, USA; 3 Department of Medicine, Division of Occupational Medicine, University of California, San Francisco, Building 30, 5th floor, San Francisco General Hospital, 1001 Potrero Avenue, San Francisco, CA 94110, USA; 4 Department of Orthopaedics, Division of Bioengineering, University of Colorado at Denver and Health Sciences Center, 12800 E. 19^th ^Ave., RC1 N, Room 2103, Mailstop 8343, PO Box 6511, Aurora, CO 80045, USA

## Abstract

Understanding the changes in collagen and proteoglycan content of cartilage due to physical forces is necessary for progress in treating joint disorders, including those due to overuse. Physical forces in the chondrocyte environment can affect the cellular processes involved in the biosynthesis of extracellular matrix. In turn, the biomechanical properties of cartilage depend on its collagen and proteoglycan content. To understand changes due to physical forces, this study examined the effect of 80 cumulative hours of *in vivo *cyclical joint loading on the cartilage content of proteoglycan and collagen in the rabbit metacarpophalangeal joint. The forepaw digits of six anesthetized New Zealand White adult female rabbits were repetitively flexed at 1 Hz with an estimated joint contact pressure of 1 to 2 MPa. Joints were collected from loaded and contralateral control specimens, fixed, decalcified, embedded, and thin-sectioned. Sections were examined under polarized light microscopy to identify and measure superficial and mid zone thicknesses of cartilage. Fourier Transform Infrared microspectroscopy was used to measure proteoglycan and collagen contents in the superficial, mid, and deep zones. Loading led to an increase in proteoglycan in the cartilage of all six rabbits. Specifically, there was a 46% increase in the cartilage deep zone (*p *= 0.003). The collagen content did not change with loading. Joint loading did not change the superficial and mid zone mean thicknesses. We conclude that long-term (80 cumulative hours) cyclical *in vivo *joint loading stimulates proteoglycan synthesis. Furthermore, stimulation is localized to cartilage regions of high hydrostatic pressure. These data may be useful in developing interventions to prevent overuse injuries or in developing therapies to improve joint function.

## Introduction

Extracellular matrix composition dictates the mechanical properties of cartilage. Proteoglycan and collagen are two important structural components of the cartilage extracellular matrix [1,2]. The highly anionic glycosaminoglycan (GAG) component of proteoglycan provides hydration and swelling pressure to the tissue and enables it to resist compressive forces. The difference between the ionic composition of the cartilage matrix and the cartilage interstitial fluid gives rise to the osmotic pressure that is always present in the extracellular matrix, even in cartilage that is unloaded [3]. Specifically, the negative fixed charges on the GAGs control the concentration of mobile ions in cartilage [4]. Because an increase in the concentration of mobile ions increases the hydrostatic pressure of cartilage, there is a direct relation between fixed charged density and the mechanical properties of cartilage, particularly stiffness [5]. Collagen fibrils confine the proteoglycan expansion and provide the extracellular matrix with tensile strength [6,7].

Cartilage is a non-homogeneous tissue. It can be divided into three distinct zones based on collagen fibril orientation [8]. The superficial zone is characterized with the fibrils aligned tangentially to the surface, the mid zone shows random alignment, whereas the deep zone contains fibrils that are oriented perpendicularly to the surface [9]. The distribution of proteoglycan and collagen varies with depth in different cartilage zones and is responsible for the load-carrying capability of cartilage. Remodeling of cartilage, as well as degradation of proteoglycans and/or the collagen fibrils, not only alters the chemical makeup of cartilage but also changes its mechanical properties. The cellular processes involved can be affected by physical forces in the chondrocyte environment as demonstrated by studies that have examined the biosynthetic response of articular cartilage explants to *in vitro *loading [10-14]. However, the biosynthetic response of cartilage to physiological loading within intact joints is not clear.

To investigate this response, we have developed a rabbit model of *in vivo *cyclical joint loading [15]. After chronic exposure (80 cumulative hours), loaded joints were prepared as thin sections to evaluate localization of extracellular matrix changes. The thicknesses of specific cartilage zones were measured with polarized light microscopy (PLM), which uses the birefringence of collagen to visualize fiber alignment [9]. Changes in chemical composition in the superficial, mid, and deep zones were examined using Fourier Transform Infrared (FTIR) microspectroscopy. FTIR microspectroscopy is a novel non-destructive method for visualizing the spatial distribution and the amount of chemical constituents in thin tissue sections [16]. Absorption peaks in an infrared spectrum represent a fingerprint of the sample under study. Infrared spectroscopy combined with microscopy can yield molecular information at the microscopic level. Our hypothesis is that physiologic, *in vivo *cyclical joint loading alters the regional chemical composition of cartilage, specifically proteoglycan.

## Materials and methods

### Joint loading

A novel *in vivo *rabbit model of repetitive joint flexion and loading was developed to simulate hand activities associated with the workplace [15]. All procedures received prior approval and oversight from the University of California's Care and Use of Animals Committee and institutional approval. The digits of adult female New Zealand White rabbits (*n *= 6) were repetitively flexed and loaded. Loading was performed with the rabbits under anesthesia. A Grass-Telefactor stimulator (Grass Technologies, West Warwick, RI, USA) was used to excite the flexor digitorum profundus (FDP) muscle of the experimental limb at 1 Hz, causing the digits to flex. A light-weight finger cuff was attached to the third digit and connected to a load cell. The stimulator voltage was adjusted to achieve an equivalent to 17.5% of the maximum muscle force of this type and size of rabbit (preliminary data, *n *= 4 rabbits). This stimulation setting resulted in the load cell measurement of 0.42 N peak force at the third digit tip. Applying free-body analysis to our cyclical joint loading protocol, we estimated that the joint contact force is approximately 3 N and the 'nominal' joint contact pressure is between 1 and 2 MPa. Loading was carried out for 80 cumulative hours in 2-hour increments, 3 days a week for 14 weeks. The contra-lateral limb (control) was neither stimulated nor loaded.

Once the loading was completed, the rabbits were euthanized and the metacarpophalangeal (MCP) joints of both limbs were removed, fixed in formalin, decalcified, embedded in paraffin, and sectioned in the sagittal plane. One section (7 μm) from each joint was placed onto an infrared-reflecting microscope slide (MirrIR low-e microscope slides; Kevley Technologies, Chesterland, OH, USA) for FTIR analyses. Three sections from each joint were placed onto Starfrost slides (Fisher Scientific International, Hampton, NH, USA) for PLM.

### FTIR analyses

The FTIR data were collected at 16-cm^-1 ^resolution using a mid-infrared Michelson-type step-scan interferometer (ThermoNicolet 870; Thermo Electron Corporation, Waltham, MA, USA) coupled to an ImageMax infrared microscope (Thermo Electron Corporation) with a 64 × 64-pixel mercury cadmium telluride focal plane array detector [17] under N_2 _purge. All FTIR imaging was carried out at the Musculoskeletal and Quantitative Imaging Research Center, University of California (San Francisco, CA, USA). Information on the amounts and distribution of proteoglycan and collagen was collected in a 200 × 100 μm region of interest (ROI). The 100-μm depth was found previously to cover the entire uncalcified cartilage of the rabbit MCP joint [15]. The FTIR images were baseline-subtracted and background-corrected. A standard paraffin spectrum was subtracted from the spectra to account for any changes in the spectra due to the embedding medium. The paraffin spectrum was collected from a 7-μm-thick section of the same paraffin as used in the embedding medium. The amount of proteoglycan was measured as the mean integrated area of the sugar peak (1,185 - 960 cm^-1^) in the ROI defined above for each FTIR image [18]. The amount of collagen was measured as the mean integrated area of the amide I peak (1,710 - 1,595 cm^-1^) in the same ROIs [18]. All FTIR data processing was performed using the Isys software package version 2.1 R1247 (Spectral Dimensions, Inc., Olney, MD, USA).

### Polarized light microscopy

Sections on slides for PLM were immersed in xylene for three cycles of 5 minutes each at 20°C to remove paraffin, which interferes with analysis. The sections were unstained and covered with a coverslip using xylene-based mounting medium (Cytoseal; Richard-Allan Scientific, Kalamazoo, MI, USA). Data were collected using a light microscope fitted with polarizers (Axioskop2; Carl Zeiss, Göttingen, Germany) and connected to a digital CCD (charge-coupled device) camera (Axiocam; Carl Zeiss). Specimens were transilluminated by polarized light. A × 20 objective was used for digital image collection. Axiovision software version 3.1 (Carl Zeiss) was used for camera control and digital image collection. During data collection, the joint articular surface for each section was aligned 45° to the polarizer axis to achieve a maximum in light intensity which is dependent upon the angle of the specimen relative to the axis of the cross polarizers. Digital images were captured. The superficial and mid zone mean thicknesses of rabbit MCP cartilage were measured in a 300 × 100 μm ROI positioned on the palmar surface of the metacarpal bone, a location used for previous studies [15]. The superficial and mid zones were outlined manually, and the areas of the zones were measured. The mean thicknesses for the superficial and mid zones were calculated as the area divided by the width.

### Proteoglycan distribution

Zonal variation of the amounts of proteoglycan and collagen was determined. Using the data from PLM, thicknesses corresponding to the superficial, mid, and deep zones were assigned to each FTIR dataset. Based on PLM analysis, the superficial zone was designated from the joint surface (at 0 μm) to 8 μm from the joint surface. The mid zone was from 8 to 53 μm, and the deep zone was from 53 to 100 μm [9]. The amounts of proteoglycan and collagen were calculated for each cartilage zone.

### Statistical analyses

To identify changes with loading in the biochemical content of cartilage, the outcome measures were mean integrated value of total proteoglycan in the full cartilage thickness and mean integrated value of total collagen in the full cartilage thickness. To identify changes with loading in the zonal thickness, the outcome measures were cartilage superficial zone mean thickness and cartilage mid zone mean thickness. To identify depth-specific changes in proteoglycan due to loading, the outcome measures were mean integrated value of proteoglycan in the superficial zone of cartilage, mean integrated value of proteoglycan in the mid zone of cartilage, and mean integrated value of proteoglycan in the deep zone of cartilage.

Two-tailed, paired Student *t *tests were used to compare control and loaded specimens (Sigma Plot version 8.1; Systat Software, Inc., Richmond, CA, USA). The statistical significance level alpha (α) was 0.05.

## Results

### Proteoglycan and collagen content in the total uncalcified cartilage

The amount of proteoglycan increased in the loaded joint compared with the control joint for all six rabbits (Table 1). The mean (± standard deviation [SD]) of integrated proteoglycan peak values of the control joints was 4.4 (± 1.9), which increased to 6.5 (± 2.4) for the loaded joints (*p *= 0.03). The mean percentage increase in proteoglycan due to loading was 64%; the median was 29.5%. A control rabbit was housed under identical conditions but without FDP stimulation or anesthesia. No difference in the amount of proteoglycan was observed between right and left joints of the limbs of this control rabbit (data not shown).

Collagen content was also measured using FTIR (Table 2). The mean (± SD) of integrated collagen peak values of the control joints was 35.3 (± 5.3) and for the loaded joints was 33.5 (± 3.8); the difference was not significant (*p *= 0.20).

The amount of proteoglycan in each ROI was normalized to the collagen content (Table 3). The mean (± SD) of normalized proteoglycan of the control joints was 0.12 (± 0.06), which increased to 0.19 (± 0.07) for the loaded joints, a statistically significant difference (*p *= 0.03). The mean percentage increase in normalized proteoglycan due to loading was 83%; the median was 35%. There was no difference between the right and left limbs of the non-treated control rabbit after normalizing to collagen.

### Superficial and mid zone thicknesses

**Figure 1:**
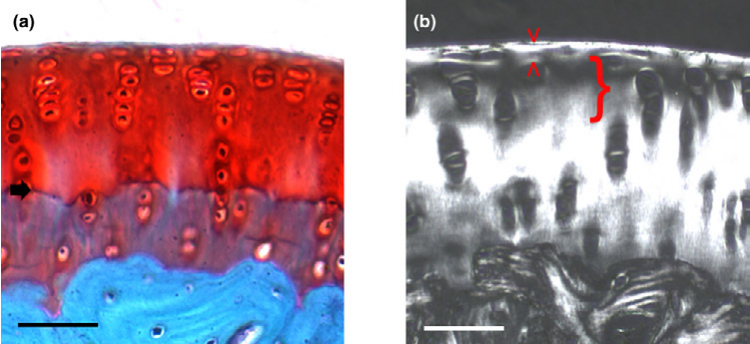
Microscopic images of joint cartilage from region of interest. **(a) **Rabbit MCP joint sections were stained with iron hematoxylin, safranin O, and fast green. This combination of stains identifies the uncalcified cartilage (red), calcified cartilage (dark red), and bone (blue-green). The tidemark (arrow) is apparent and marks the division between the uncalcified and calcified cartilage. **(b) **Unstained sections were imaged under polarized light microscopy. The superficial zone is indicated by the two arrows at the articular surface. The bracket encloses the mid zone, which due to the anisotropic arrangement of collagen fibers does not exhibit birefringence. Bars = 100 μm.

The superficial and mid zone thicknesses of rabbit MCP joint cartilage were measured using PLM (Figure 1; Table 4). The mean (± SD) superficial zone thicknesses for the control and loaded joints were 8.11 μm (± 1.2) and 8.3 μm (± 1.7), respectively. The mean (± SD) mid zone thicknesses for the control and loaded joints were 44.03 μm (± 7.9) and 45.72 μm (± 8.2), respectively. There were no statistically significant differences between the thicknesses of the control versus the loaded joints for the superficial (*p *= 0.77) and mid (*p *= 0.73) zones.

### Zonal variation of proteoglycan

**Figure 2:**
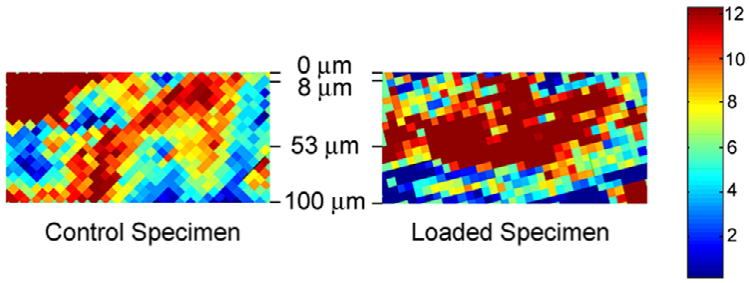
Representative Fourier Transform Infrared maps of proteoglycan in the unloaded and loaded joints of one rabbit. The superficial zone was designated from the joint surface (at 0 μm) to 8 μm from the joint surface. The mid zone was from 8 to 53 μm, and the deep zone was from 53 to 100 μm. Each image is 100 μm deep and 200 μm wide. Integrated proteoglycan peak absorbance values are pseudo-colored; red indicates that more proteoglycan is present and blue indicates that less proteoglycan is present. These maps were typical of all rabbits.

Mean zone thicknesses were applied to the FTIR maps to measure the zonal variation of proteoglycan (Figure 2). The mean (± SD) of integrated proteoglycan peak values (normalized to collagen content) of the control joints in the superficial zone of cartilage was 0.16 (± 0.09) and was 0.18 (± 0.10) for the loaded joints. The difference between the control and loaded superficial zone proteoglycans was not significant (*p *= 0.60). In the mid zone, the mean (± SD) of integrated proteoglycan peak values (normalized to collagen content) of the control joints was 0.14 (± 0.06) and 0.20 (± 0.09) for the loaded joints. The difference between the control and loaded mid zone proteoglycan was not significant (*p *= 0.20).

However, in the deep zone, the amount of proteoglycan increased significantly with loading (*p *= 0.003). The mean (± SD) of integrated proteoglycan peak values of the control joints (normalized to collagen content) was 0.11 (± 0.07) and 0.17 (± 0.07) for the loaded joints. In all six rabbits, deep zone proteoglycan increased with loading. The mean of percentage increase in deep zone proteoglycan between control and loaded joints was 76%; the median was 46% (Table 5).

## Discussion

This is the first study to measure an increase in proteoglycan content due to physiologic *in vivo *cyclical joint loading. Using microscopic FTIR spectroscopy, we demonstrate that the increase of proteoglycan is localized to the deep zone of cartilage, indicating that 80 cumulative hours of physiological joint loading leads to stimulation of proteoglycan synthesis in a spatially specific manner.

A number of *in vitro *studies have measured the metabolic effects of cartilage loading using unconfined compression [10-14,19]. Sah *et al*. [10], Larsson *et al*. [11], and Parkkinen *et al*. [19] have observed increased proteoglycan biosynthesis in bovine articular cartilage explants with dynamic compression. In contrast, others have found a decrease in proteoglycan biosynthesis with unconfined compression *in vitro *[12-14]. These difference can be attributed to both the stimulatory effects (fluid pressure, convective transport) [20,21] and inhibitory effects (matrix consolidation due to large strains) that occur in unconfined compression [14]. Although compressive loading of cartilage explants provides valuable insights, it does not model true load-carrying in physiologic joint function. From *in vitro* experiments, it has been proposed that stimulation of proteoglycan synthesis is associated with tissue regions having high interstitial fluid flow [22]. However, the fluid flow and lateral expansion of the cartilage *in vivo* are limited by the surrounding cartilage and bone [23], and it is the pressurization of the interstitial fluid due to these boundaries that exerts the effective load on cartilage [24,25]. Ikenoue *et al.*[26] have demonstrated increased aggrecan expression and synthesis in high-density cultures of human chondrocytes that received intermittent hydrostatic pressure of 1, 5, or 10 MPa at 1 Hz. They also demonstrate increased expression of type II collagen; however, collagen protein increased only at high pressure, 10 MPa, and weakly at 5 MPa. The findings of the low-pressure experiments by Ikenoue *et al.*[26] are most relevant to the present study because we estimate that the hydrostatic pressure generated within the deep zone of the MCP joints in our experiments is approximately 1 to 2 MPa. Our findings support the hypothesis that increased levels of fluid pressure stimulate proteoglycan biosynthesis.

In our study, the increase in proteoglycans was localized to the deep zone of cartilage. This is in agreement with a previous finding *in vitro *[27] in which proteoglycan synthesis increased by 102% to 114% in the deep zone after 4 hours of dynamic unconfined loading. The same study, however, found that the stimulatory effect was decreased after 8 hours. This loss of effect may be due to the loss of hydrostatic pressure over time. With unconfined compression *in vitro*, the fluid can flow out from the explant edges, and this may lead to lower hydrostatic pressure and thus lower proteoglycan production than exists in vivo under the same frequencies and amplitudes of loading.

Our results support the proposed hypothesis that high hydrostatic pressure as produced in cyclical joint loading promotes matrix synthesis in cartilage [28]. Aggrecan gene expression may be upregulated by the hydrostatic pressure of physiologic range that is exerted by cyclical *in vivo *loading in the deep zone of cartilage. The deep zone of cartilage is loaded primarily under hydrostatic pressure and experiences little fluid flow *in vivo *[29]. Studies by Hall *et al*. [30] and Parkkinen *et al*. [19] demonstrate *in vitro *that hydrostatic pressure increases proteoglycan synthesis in cartilage explants. Chondrocytes are the sole regulators of cartilage biosynthetic activity and respond to external mechanical stimuli such as hydrostatic pressure and fluid shear [10,28,31]. Stretch-activated ion channels might be part of the transduction pathway of repetitive forces [32,33]. We hypothesize that the increase in proteoglycan in the cartilage deep zone is due to the localized increase in hydrostatic pressure caused by cyclical *in vivo *loading that leads to signal transduction within the resident chondrocytes.

Our experiment is long-term (>3 months), and therefore we expect all components of proteoglycan synthesis to have been upregulated. For example, the genes required for aggrecan synthesis and post-translational modification include the protein core [34], xylosyl tranferase [35], and sulfotransferases [36]. Recently, the transcription rates of genes for sulfotransferases C4ST1, C4ST2, and C6ST1 all have been shown to increase with *in vitro *dynamic compression [37].

With FTIR microscpectroscopy, no significant change in the amount of collagen is found with 80 hours of cumulative loading. However, this is expected given the extremely long half-life (200 to 400 years) reported for cartilage collagen [38]. The turnover rate of collagen is much slower than that of aggrecan [39,40]. It should be noted, however, that alterations to the collagen network organization may have occurred without changes in the amount of collagen after loading. Arokoski *et al*. [41] observed that, although joint loading through long-distance running in dogs generally did not significantly change the thickness of cartilage zones, birefringence intensity decreased in the superficial zone; the authors attributed this decrease to a localized loss of collagen network organization. Because we were unable to measure birefringence intensity, we cannot make detailed conclusions on collagen network organization in this study.

Our study does not determine the minimum number of hours of loading required to detect an increase in proteoglycan, nor whether this increase is sustained, because all joints were loaded for 80 cumulative hours and a study with multiple time points would be required. Also, because a single loading pattern was used, we cannot draw conclusions about the effect of *in vivo *loading on proteoglycan at other forces and frequencies. Future studies will test other durations, peak forces, and frequencies of *in vivo *joint loading.

## Conclusion

**Figure 3:**
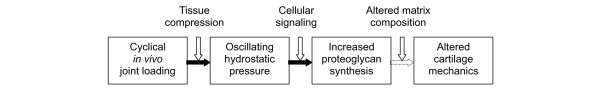
A proposed mechanism by which cyclical loading leads to changes in articular cartilage mechanical properties. Hydrostatic pressure of cartilage tissue is increased through compression caused by cyclical *in vivo *joint loading. With loading at physiological frequencies and amplitudes, changes in cellular signaling pathways lead to a detectable increase in proteoglycan synthesis. Changes in chemical composition of cartilage extracellular matrix eventually lead to changes in mechanical properties of the tissue in general.

We conclude that cyclical *in vivo *joint loading increases the proteoglycan content of the cartilage deep zone via signal transduction stimulated by increased hydrostatic pressure (Figure 3). This is clinically significant because the biomechanical properties of cartilage, and therefore its function, depend to a large extent on its ability to maintain hydration and tissue thickness under mechanical stresses with normal physiological loading. Proteoglycans provide the osmotic resistance necessary for cartilage to resist compressive loads.

An increase in the amount of cartilage proteoglycan could indicate a healthy mode of joint response to loading in which chondrocytes detect and respond to changes in their mechanical environment by increasing proteoglycan biosynthesis. However, the mechanical effect of changing the ratio of proteoglycan to collagen was not tested and the contents of other matrix proteins were not measured. Nonetheless, using our *in vivo *joint loading model, we will be able to test different loading patterns to determine thresholds of healthy and damaging loading. These thresholds would be useful in designing force and frequency patterns to prevent overuse joint injuries. Knowledge of the effects of loading patterns may also benefit tissue engineering strategies and post-surgical rehabilitation.

## Abbreviations

FDP = flexor digitorum profundus; FTIR = Fourier Transform Infrared; GAG = glycosaminoglycan; MCP = metacarpophalangeal; PLM = polarized light microscopy; ROI = region of interest; SD = standard deviation.

## Competing interests

The authors declare that they have no competing interests.

## Authors' contributions

ES carried out the FTIR data acquisition and analysis, performed the statistical analyses, and drafted the manuscript. HL carried out the PLM data acquisition and analysis and helped draft the manuscript. SM provided FTIR equipment and expertise and helped edit the manuscript. DR assisted with the *in vivo *model and helped edit the manuscript. KK conceived of and supervised the project, supervised all data collection and analysis, and edited the manuscript. All authors read and approved the final manuscript.

## Figures and Tables

**Table 1:** Proteoglycan content in cartilage of cyclically loaded rabbit metacarpophalangeal joints and their contra-lateral controls

Rabbit no.	Control PG^a ^(absorbance)	Loaded PG (absorbance)	Difference
1	2.1	7.0	4.9
2	2.1	2.6	0.5
3	6.5	10.3	3.8
4	5.2	6.5	1.3
5	4.6	6.0	1.4
6	6.3	6.9	0.6

Mean	4.4	6.5	2.1
SD	1.9	2.4	1.8
Significance^b^			0.03

^a^PG content was measured as the mean integrated PG peak (1,185 - 960 cm^-1^) value from the Fourier Transform Infrared spectra; ^b^statistical significance was calculated using the two-tailed, paired Student *t *test (α = 0.05). PG, proteoglycan; SD, standard deviation.

**Table 2:** Collagen content in the cartilage of cyclically loaded rabbit metacarpophalangeal joints and their contra-lateral controls

Rabbit no.	Control collagen^a ^(absorbance)	Loaded collagen (absorbance)	Difference
1	37.8	31.8	-6.0
2	29.8	28.2	-1.6
3	31.1	33.0	1.9
4	42.6	38.3	-4.3
5	31.0	32.1	1.1
6	39.5	37.5	-2.0

Mean	35.3	33.5	-1.8
SD	5.3	3.8	3.0
Significance^b^			>0.05

^a^Collagen content was measured as the mean integrated collagen peak (1,710 - 1,595 cm^-1^) value from the Fourier Transform Infrared spectra; ^b^statistical significance was calculated using the two-tailed, paired Student *t *test (α = 0.05). SD, standard deviation.

**Table 3:** Normalized proteoglycan content in the cartilage of cyclically loaded rabbit metacarpophalangeal joints and their contra-lateral controls

Rabbit no.	Normalized^a ^PG content (control)	Normalized PG content (loaded)	Difference
1	0.05	0.22	0.17
2	0.07	0.09	0.02
3	0.21	0.31	0.10
4	0.12	0.17	0.05
5	0.14	0.18	0.04
6	0.16	0.18	0.02

Mean	0.12	0.19	0.07
SD	0.06	0.07	0.06
Significance^b^			0.03

^a^The normalized values were obtained by dividing the value of PG content from each joint by the value of the collagen content of that joint; ^b^statistical significance was calculated using the two-tailed, paired Student *t *test (α = 0.05). PG, proteoglycan; SD, standard deviation.

**Table 4:** Zone thicknesses of articular cartilage in cyclically loaded rabbit metacarpophalangeal joints and their contra-lateral controls

	Superficial zone thickness (μm)			Mid zone thickness (μm)		
		
Rabbit no.	Control	Loaded	Difference	Control	Loaded	Difference
1	8.8	9.7	0.9	51.3	55.9	4.6
2	7.9	8.9	1.0	38.2	40.7	2.5
3	10.0	9.0	-1.1	50.3	49.6	-0.7
4	7.1	8.7	1.5	51.7	34.7	-17.0
5	7.5	8.5	1.0	38.5	40.9	2.4
6	7.2	5.0	-2.3	34.1	52.4	18.4

Mean	8.1	8.3	0.2	44.0	45.7	1.7
SD	1.1	1.7	1.5	7.9	8.1	11.3
Significance^a^			>0.05			>0.05

^a^Significance was calculated using the two-tailed, paired Student *t *test (α = 0.05). SD, standard deviation.

**Table 5:** Zonal distribution of proteoglycan

	Normalized PG in superficial zone^a^			Normalized PG in mid zone^b^			Normalized PG in deep zone^c^		
			
Rabbit no.	Control	Loaded	Difference	Control	Loaded	Difference	Control	Loaded	Difference
1	0.14	0.20	0.06	0.06	0.29	0.23	0.03	0.11	0.08
2	0.05	0.01	-0.04	0.09	0.06	-0.02	0.06	0.09	0.03
3	0.27	0.33	0.06	0.22	0.31	0.09	0.19	0.28	0.09
4	0.27	0.18	-0.08	0.13	0.19	0.05	0.07	0.14	0.07
5	0.18	0.17	-0.01	0.17	0.16	-0.01	0.14	0.18	0.04
6	0.06	0.17	0.11	0.15	0.15	0.00	0.18	0.22	0.04

Mean	0.16	0.18	0.02	0.14	0.20	0.06	0.11	0.17	0.06
SD	0.09	0.10	0.07	0.06	0.09	0.10	0.07	0.07	0.03
Significance^d^			>0.05			>0.05			0.003

^a^Superficial zone of cartilage is from 0 to 8 μm; ^b^mid zone is from 8 to 53 μm; ^c^deep zone is from 53 to 100 μm; the zone thicknesses were determined by polarized light microscopy; ^d^significance was calculated using the two-tailed, paired Student *t *test (α = 0.05). PG, proteoglycan; SD, standard deviation.
